# Targeting of PI3K/AKT/mTOR pathway to inhibit T cell activation and prevent graft-versus-host disease development

**DOI:** 10.1186/s13045-016-0343-5

**Published:** 2016-10-20

**Authors:** Mª Carmen Herrero-Sánchez, Concepción Rodríguez-Serrano, Julia Almeida, Laura San Segundo, Susana Inogés, Ángel Santos-Briz, Jesús García-Briñón, Luis Antonio Corchete, Jesús F. San Miguel, Consuelo del Cañizo, Belén Blanco

**Affiliations:** 1 Servicio de Hematología, Hospital Universitario de Salamanca, Paseo de San Vicente 58-182, 37007 Salamanca, Spain; 2 Instituto de Investigación Biomédica de Salamanca (IBSAL), Paseo de San Vicente 58-182, 37007 Salamanca, Spain; 3 Centro de Investigación del Cáncer, Universidad de Salamanca, Campus Miguel de Unamuno, 37007 Salamanca, Spain; 4 Servicio de Citometría, Centro de Investigación del Cáncer, Universidad de Salamanca, Campus Miguel de Unamuno, 37007 Salamanca, Spain; 5 Laboratorio de Inmunoterapia, Clínica Universidad de Navarra, Avda. Pío XII 55, 31008 Pamplona, Spain; 6 Departamento de Patología, Hospital Universitario de Salamanca, Paseo de San Vicente 58-182, 37007 Salamanca, Spain; 7 Departamento de Biología Celular y Patología, Facultad de Medicina, Campus Miguel de Unamuno, 37007 Salamanca, Spain; 8 Clínica Universidad de Navarra, Centro de Investigación Médica Aplicada, Instituto de Investigación Sanitaria de Navarra, Avda. Pío XII 55, 31008 Pamplona, Spain

**Keywords:** Hematopoietic stem cell transplantation, Graft-versus-host disease, T cell, PI3K/AKT/mTOR pathway, PI3K inhibitor

## Abstract

**Background:**

Graft-versus-host disease (GvHD) remains the major obstacle to successful allogeneic hematopoietic stem cell transplantation, despite of the immunosuppressive regimens administered to control T cell alloreactivity. PI3K/AKT/mTOR pathway is crucial in T cell activation and function and, therefore, represents an attractive therapeutic target to prevent GvHD development. Recently, numerous PI3K inhibitors have been developed for cancer therapy. However, few studies have explored their immunosuppressive effect.

**Methods:**

The effects of a selective PI3K inhibitor (BKM120) and a dual PI3K/mTOR inhibitor (BEZ235) on human T cell proliferation, expression of activation-related molecules, and phosphorylation of PI3K/AKT/mTOR pathway proteins were analyzed. Besides, the ability of BEZ235 to prevent GvHD development in mice was evaluated.

**Results:**

Simultaneous inhibition of PI3K and mTOR was efficient at lower concentrations than PI3K specific targeting. Importantly, BEZ235 prevented naïve T cell activation and induced tolerance of alloreactive T cells, while maintaining an adequate response against cytomegalovirus, more efficiently than BKM120. Finally, BEZ235 treatment significantly improved the survival and decreased the GvHD development in mice.

**Conclusions:**

These results support the use of PI3K inhibitors to control T cell responses and show the potential utility of the dual PI3K/mTOR inhibitor BEZ235 in GvHD prophylaxis.

**Electronic supplementary material:**

The online version of this article (doi:10.1186/s13045-016-0343-5) contains supplementary material, which is available to authorized users.

## Background

Allogeneic hematopoietic stem cell transplantation (allo-HSCT) remains the only curative option for many hematologic malignancies. Unfortunately, a serious complication is frequently developed after allo-HSCT: graft-versus-host disease (GvHD). GvHD occurs when donor T lymphocytes recognize as foreign and destroy patient’s healthy tissues. Despite the immunosuppressive regimens administered, GvHD remains the major cause of morbidity and mortality after allo-HSCT. Thus, new therapeutic strategies are needed.

One of the key signaling pathways involved in T cell activation and function is phosphatidylinositol 3-kinase/AKT/mammalian target of rapamycin (PI3K/AKT/mTOR) [1]. This pathway controls numerous cellular processes, including proliferation, survival, migration, and metabolism [2]. In particular, PI3K activation in T cells promotes survival [3] and cell cycle progression [4], modulates differentiation [5, 6], and controls the acquisition of effector and memory phenotypes [7]. Thus, inhibitors of PI3K/AKT/mTOR pathway can interfere with T cell activation and function.

The use of PI3K/AKT/mTOR inhibitors has been scantily explored in the allo-HSCT context. Only the utility of the mTORC1 inhibitor rapamycin (Sirolimus) has been extensively studied, providing promising results [8]. In addition, it has been suggested that the beneficial effects observed in patients with chronic GvHD treated with tyrosine kinase inhibitors could be due, in part, to their ability to inhibit PI3K signaling in T cells [9]. However, few studies have evaluated the immunosuppressive effect of PI3K inhibitors on T lymphocytes [10–12] and their ability to prevent GvHD development [13, 14]. Herein, we have analyzed the effects of two novel antitumor drugs, the pan-class I PI3K inhibitor BKM120 and the dual PI3K/mTOR inhibitor BEZ235, on T cell activation and evaluated the utility of BEZ235 in a murine model of GvHD.

## Methods

### Drugs

BEZ235 was kindly provided by Novartis Pharma (Basel, Switzerland). BKM120 was purchased from Selleck Chemicals (Houston, TX, USA). For in vitro studies, BKM120 and BEZ235 were reconstituted in DMSO at 10 mM and stored frozen at −20 °C until use. For in vivo assays, BEZ235 solution was prepared fresh before administration. In brief, BEZ235 was dissolved in one volume of N-methyl-2-pyrrolidone (Sigma-Aldrich, St. Louis, MO) and then nine volumes of polyethylene glycol 300 (Sigma-Aldrich) were added. The application volume was 10 ml/kg body weight.

### Cell isolation and culture

Peripheral blood mononuclear cells (PBMCs) were isolated from buffy coats of volunteer healthy donors by density gradient centrifugation using Ficoll–Paque solution (GE Healthcare Bio-Sciences, Uppsala, Sweden). Buffy coats were provided by the Centro de Hemoterapia y Hemodonación de Castilla y León, after written informed consent obtention. The research was approved by the Clinical Research Ethics Committee (CEIC) of “*Area de Salud de Salamanca*” (2012/11/132).

For Western blot analysis, PBMCs were allowed to adhere to tissue culture flasks (Corning, NY, USA) O/N at 37 °C and thereafter, non-adherent cells (T cell-enriched PBMCs) were collected. For cell cycle, apoptosis, and cytokine secretion assays, T cells were isolated from PBMCs by immunomagnetic selection, using the Pan T Cell Isolation Kit (Miltenyi Biotec, Bergisch Gladbach, Germany). The purity of isolated populations was routinely >95 %.

PBMCs or isolated T cells were cultured in well plates (Greiner Bio-One, Frickenhausen, Germany) at a density of 1 × 10^6^ cells/ml in RPMI 1640 medium supplemented with 2 mM l-glutamine, 100 U/ml penicillin, 100 μg/ml streptomycin (all from Gibco-Invitrogen, Paisely, UK), and 10 % human AB serum (Sigma-Aldrich). Cells were stimulated or not with plate-bound anti-CD3 (5 μg/ml) and soluble anti-CD28 (2.5 μg/ml) monoclonal antibodies (mAbs) (BD Biosciences, San Jose, CA, USA) and treated with different doses of BKM120 or BEZ235 (0–10 μM).

### Western blot analysis

Unstimulated or stimulated T cell-enriched PBMCs were treated with different concentrations of BKM120 or BEZ235. After 48 h, cells were lysed and cell extracts were electrophoresed, transferred onto PVDF membrane (Millipore, Bedford, MA, USA), and immunoblotted with antibodies against caspase 3, phosphorylated AKT (p-AKT) (T308 and S473), total AKT, phosphorylated 4E-BP1 (p-4E-BP1) (T37/46), total 4E-BP1, phosphorylated RPS6 (p-RPS6) (S235/236), total RPS6, phosphorylated p38 MAPK (p-p38) (T180/Y182), total p38 MAPK, phosphorylated ERK1/2 (p-ERK1/2) (T202/Y204) (all from Cell Signaling Thechnology®, Leiden, Netherlands), or total ERK2 (Santa Cruz Biotechnology, Heidelberg, Germany); antibodies to GAPDH (Cell Signaling Thechnology®) and calnexin (Enzo® Life Science, Plymouth Meeting, PA, USA) were used as loading controls. Anti-rabbit or anti-mouse antibodies conjugated to horseradish peroxidase (GE Healthcare, Buckinghamshire, UK) were used as secondary antibodies. Proteins were visualized with an ECL detection system (GE Healthcare).

### Proliferation assays

PBMCs were stained with PKH-67 green fluorescent dye (Sigma-Aldrich) following manufacturer’s instructions. Thereafter, PKH-stained cells were seeded in the absence or in the presence of anti-CD3/anti-CD28 mAbs as described above and treated with different concentrations of the drugs. After 5 days, cells were stained with 7-amino-actinomycin D (7AAD) and anti-CD3-APC and acquired in a FACSCalibur flow cytometer (all from BD Biosciences). Percentage of proliferating T cells (CD3^+^PKH^low^) was calculated using the Infinicyt software (Cytognos, Salamanca, Spain).

### Cell cycle analysis

Unstimulated or anti-CD3/anti-CD28 stimulated isolated T cells were cultured in the presence of different concentrations of BKM120 or BEZ235. After 4 days, cells were stained with propidium iodide, using the CycleTEST™ PLUS DNA Reagent Kit (BD Biosciences) and acquired on a FACSCalibur flow cytometer. The distribution of cells along the cell cycle phases was analyzed using ModFit LT™ Macintosh program (Verity Software House, Topsham, ME, USA).

### Apoptosis assessment

Unstimulated or anti-CD3/anti-CD28 stimulated isolated T cells were cultured in the presence of different concentrations of the compounds. After 2 days, cells were stained with Annexin V-PE (BD Pharmingen™, San Diego, USA). Samples were acquired on a FACSCalibur flow cytometer and analyzed using the Infinicyt software.

### Cytokine assays

Isolated T cells were stimulated with anti-CD3/anti-CD28 mAbs in the presence of several concentrations of BKM120 or BEZ235. After 48 h, concentration of different cytokines in culture supernatants was analyzed on a FACSCalibur flow cytometer using the Human Th1/Th2 Cytokine Cytometric Bead Array (CBA) kit and BD CBA software (all from BD Biosciences).

### Immunophenotypic analysis

Unstimulated or anti-CD3/anti-CD28 stimulated PBMCs were treated with different concentrations of BKM120 or BEZ235. After 48 h, cells were stained with the following combination of mAbs: anti-CD45RA-FITC/anti-IFN-γ-PE/anti-CD8-PerCP-Cy5.5/anti-CD25-PE-Cy7/anti-Granzyme B-Alexa Fluor® 647/anti-CD4-APC-Alexa Fluor® 750/anti-CD27-Brillant Violet 421/anti-CD3-Brillant Violet 510. For intracellular staining of IFN-γ and granzyme B, brefeldin A (10 μg/ml) (Sigma-Aldrich) was added for the last 4 h prior to acquisition and the IntraStain Kit (Dako Cytomation, Denmark) was used. Acquisition was performed on a FACSCanto flow cytometer (BD) and analyzed using the Infinicyt software.

### Enzyme-linked immunospot (ELISPOT) assays

PBMCs from cytomegalovirus (CMV)-positive donors (responder cells) were stimulated with irradiated (25 Gy) PBMCs from a second donor (allogeneic cells) in a 2:1 ratio. Different doses of BKM120 or BEZ235 were added. After 96 h, responder cells were collected and cultured, in the absence of drugs, in an IFN-γ ELISpot plate (Mabtech, Nacka Strand, Sweeden): (a) in the absence of stimulation (control), (b) re-stimulated with allogeneic cells from the same donor of the primary culture, or (c) re-stimulated with CMV-pp65 recombinant protein (Miltenyi Biotec). After 36 h, ELISPOT was performed following manufacturer’s instructions. Spots corresponding to IFN-γ secreting cells were quantified using an Immunospot ELISPOT reader (CTL, Aale, Germany). The percentage of IFN-γ secreting cells was determined by subtracting, from the number of spots counted in allogeneic or CMV-pp65 re-stimulated wells, the background spots in the corresponding unstimulated (control) wells. These values were normalized with respect to those obtained from the samples pre-stimulated in the absence of drugs (0 μM) and re-stimulated with allogeneic cells or CMV-pp65, respectively, considered as 100 %.

### GvHD murine model

Female recipient Balb/c (H2d) and male donor C57BL/6 (H2b) mice (12 weeks old) were purchased from Harlan Laboratory (Carshalton, UK). Animal experiments were approved by the ethical committee of Salamanca University (N°201300004045).

Balb/c mice received total body irradiation (8.5 Gy in two fractions) from a Cs137 source and then an intravenous injection of 10 × 10^6^ C57BL/6 bone marrow cells without (BM group) or with 5 × 10^6^ splenocytes. Mice receiving splenocytes were either untreated (GvHD group) or treated orally with BEZ235 (GvHD + BEZ235 group) once a day, from day +1 to day +60 post-transplantation. Different concentrations of BEZ235 (5–50 mg/kg/day) were tested and, finally, the best results were observed at a dose of 5 mg/kg/day.

Four experiments were performed with at least two mice per group. A control mouse receiving irradiation without stem cell support (Total Body Irradiation, TBI group) was also included in each experiment.

Balb/c mice were monitored daily for survival and for the following clinical signs of GvHD: weight loss, posture (hunching), activity, fur texture and skin integrity. Each parameter received a score of 0 (normal), 1 (mild to moderate), or 2 (severe) and a clinical GvHD index was generated subsequently by summation of the five criteria scores (maximum index = 10), as previously described [15]. All moribund mice were humanely killed.

For histopathological analysis of GvHD target organs (large intestine, skin and liver), at least one animal of each group was killed in the third week post-transplantation and once treatment was completed. Tissues were fixed in 10 % neutral-buffered formalin (Sigma-Aldrich), embedded in paraffin, and sectioned and stained with hematoxylin and eosin (both from Merck KGaA, Darmstadt, Germany). Slides were examined under a BX41 light microscope and images were captured using a DP50 digital camera and the software Cell^A 2.6 (all from Olympus Optical Co. Ltd., Tokyo, Japan). Details of the scoring system are summarized in Table 1.

**Table 1:** Histologic criteria for GvHD score

Score	Skin	Liver	Large intestine
0	Normal	Normal	Normal
0.5		Focal mild portal lymphoid infiltrate	Occasional or rare necrotic cells in glands or crypts
1	Basal vacuolar change	Widespread mild portal lymphoid infiltrate	Isolated apoptotic epithelial cells, without crypt loss
2	Dyskeratotic cells in the epidermis and/or follicle, dermal lymphocytic infiltrate	Focal bile duct invasion or cellular injury	Individual crypt loss. Regeneration changes
3	Fusion of basilar vacuoles to form clefts and microvesicles	Multiple foci of bile duct injury and regeneration	Contiguous area of multiple crypt loss
4	Separation of the epidermis from the dermis	Widespread injury and destruction of bile ducts	Extensive crypt dropout with denudation of epithelium

### Statistical analysis

Most statistical analyses were performed using IBM SPSS Statistics 20 (Chicago, IL, USA). Differences between the effects of different doses of a drug and between both drugs were analyzed by the Kruskal–Wallis multiple-comparison Z value test. Pairwise comparisons were performed using the Mann–Whitney test with Bonferroni correction. Survival curves were plotted using Kaplan–Meier estimates and a log-rank test was used to compare survival rates.

For statistical analysis of weight variation and clinical scores, curves of each mouse along time from transplantation were fitted by cubic splines using the program “Compare” in the SIMFIT statistical package (http://simfit.org.uk), applying unweighted least square fitting. Areas under the curve (AUCs) from the replicate curves of mice in each group were compared by a one-way ANOVA followed by the Tukey’s post hoc test.

Statistical significance in all tests was concluded for values of *p* < 0.05.

## Results

### Effect of BKM120 and BEZ235 on PI3K/AKT/mTOR pathway

**Fig. 1:**
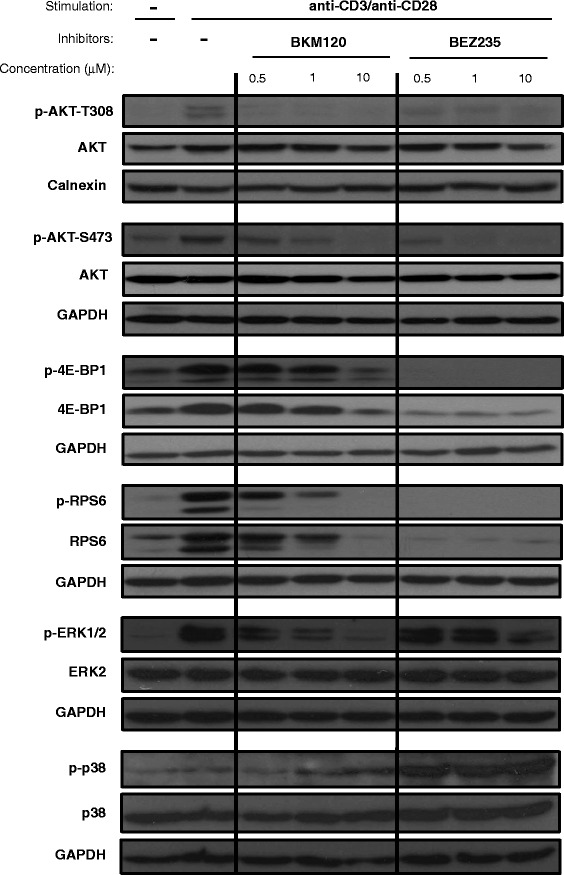
Effect of BKM120 and BEZ235 on phosphorylation of PI3K/AKT/mTOR and MAPK pathway proteins. T cell-enriched PBMCs were stimulated for 48 h with anti-CD3 and anti-CD28 mAbs in the presence of different concentrations of BKM120 or BEZ235. Analysis of phosphorylation and expression of different proteins belonging to PI3K/AKT/mTOR and MAPKs pathways was performed. Western blot representative of at least three independent experiments

First, the effect of BKM120 and BEZ235 on the phosphorylation of PI3K/AKT/mTOR pathway proteins in stimulated T cells was assessed (Fig. 1). BKM120 effectively reduced AKT phosphorylation at T308 at all the tested doses, and so did BEZ235, although with less efficacy. The treatment with both inhibitors also reduced AKT-S473 phosphorylation, observing a complete abrogation at 1 μM for BEZ235 and 10 μM for BKM120.

The amount of phosphorylated 4E-BP1 and RPS6 proteins was also diminished in the presence of both drugs. However, while this decrease was clear only at the highest concentration of BKM120 (10 μM), the lowest dose of BEZ235 (0.5 μM) was sufficient to completely abolish the presence of the phosphorylated forms of 4E-BP1 and RPS6. Of note, this complete abrogation was accompanied by the strong reduction in RPS6 and 4E-BP1 expression.

As 4E-BP1 expression can be negatively regulated by the mitogen-activated protein kinases (MAPKs) ERK and p38 [16], their phosphorylation was assessed. Both drugs reduced ERK1/2 phosphorylation on stimulated T cells. However, BKM120 (10 μM) and, especially, BEZ235 (0.5–10 μM) increased p38 phosphorylation, correlating with the reduction in RPS6 and 4E-BP1 expression (Fig. 1).

### Effect of BKM120 and BEZ235 on T cell proliferation and apoptosis induction

**Fig. 2:**
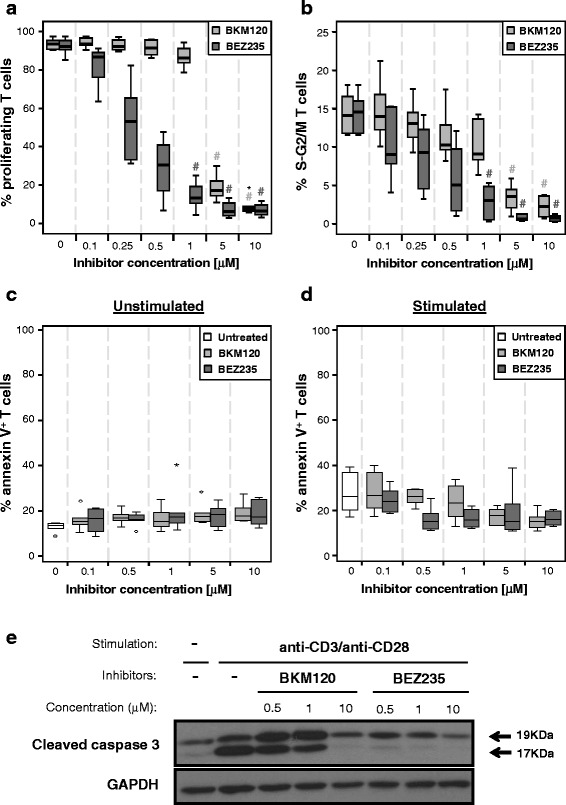
Effect of BKM120 and BEZ235 on T cell proliferation and apoptosis induction. PBMCs (**a**) or isolated T cells (**b**) were stimulated and treated with different concentrations of BKM120 or BEZ235. **a** Percentage of T cells that had undergone one or more cell divisions (CD3^+^PKH^low^) after 5 days of culture. **b** Percentage of T cells in synthesis and G2/mitosis (S-G2/M) phases after 4 days of culture. #*p* < 0.05 with respect to untreated control (0 μM). Percentage of annexin V^+^ cells among unstimulated (**c**) or stimulated (**d**) isolated T cells. Data pooled from six independent experiments*.* Outliers are represented by circles (values > 1.5 × IQR) and stars (values > 2 × 1.5 × IQR). *IQR*, interquartile range*.* **e** Western blot analysis of cleaved caspase 3 in T cell-enriched PBMCs. Results representative from three independent experiments

Thereafter, we assessed the effect of both inhibitors on stimulated T cell proliferation. The percentage of proliferating T cells significantly decreased at high concentrations of both drugs, although at low doses BEZ235 was much more effective than BKM120 (Fig. 2a).

Next, we investigated whether this reduction in proliferation was related to cell cycle arrest, to an increase in apoptosis or to both. The percentage of cells in synthesis and G2/mitosis phases significantly decreased among stimulated T cells in the presence of both inhibitors, although, at low doses, BEZ235 was more efficient than BKM120 (Fig. 2b).

Regarding apoptosis, the addition of inhibitors did not significantly change the percentage of annexin V^+^ cells, neither among unstimulated nor among stimulated T cells (Fig. 2c, d). However, the amount of cleaved caspase 3 in stimulated cells decreased in the presence of BEZ235 and at high concentrations of BKM120 (Fig. 2e).

### Effect of BKM120 and BEZ235 on T cell cytokine secretion

**Fig. 3:**
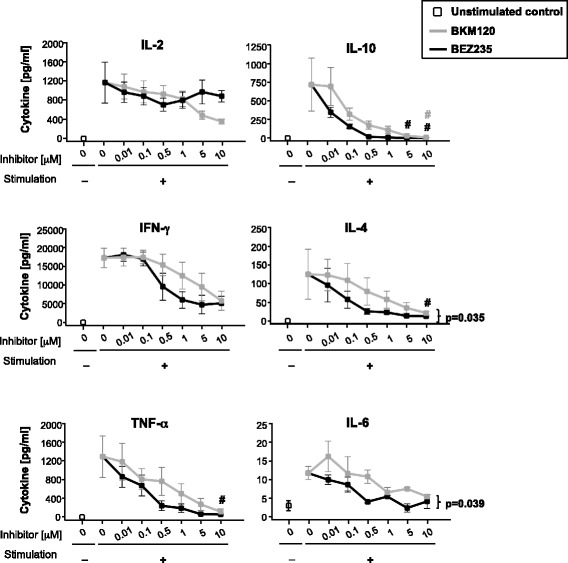
Effect of BKM120 and BEZ235 on Th1/Th2 cytokine secretion. Concentration of IL-2, IFN-γ, TNF-α, IL-4, IL-10, and IL-6 in the culture supernatant of isolated T cells stimulated in the presence of different concentrations of BKM120 or BEZ235. Concentration values corresponding to unstimulated untreated samples are also shown. Data represent the mean ± SEM of at least three independent experiments. #*p* < 0.05 with respect to stimulated untreated samples (0 μM)

Both inhibitors induced, in general, a dose-dependent decrease in Th1/Th2 cytokine secretion. The effect of BEZ235 was greater than that of BKM120 at low/intermediate doses (Fig. 3). As an exception, and despite an initial dose-dependent decrease, IL-2 concentration started a tendency to recover the levels of stimulated untreated cells at BEZ235 concentrations ≥1 μM.

### Effect of BKM120 and BEZ235 on stimulated T cell phenotype

Another interesting point was to evaluate whether the inhibitors impaired the expression of T cell activation markers. For this purpose, cell surface expression of CD25 and intracellular expression of IFN-γ and granzyme B were analyzed on CD4^+^ and CD8^+^ stimulated T cells.

**Fig. 4:**
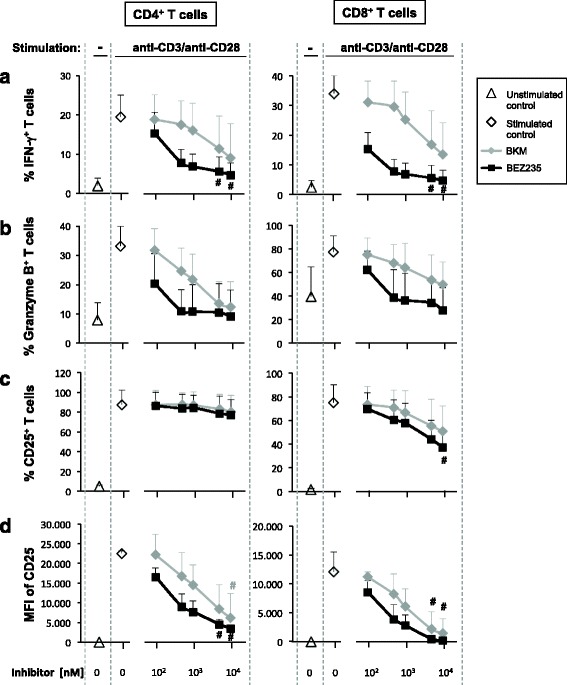
Effect of BKM120 and BEZ235 on expression of T cell activation markers. Percentage of IFN-γ^+^ (**a**), granzyme B^+^ (**b**), and CD25^+^ (**c**) cells among CD4^+^ and CD8^+^ T cells, unstimulated or stimulated in the presence of different concentrations of BKM120 and BEZ235. Data represent the mean + SD from five independent experiments. **d** MFI of CD25 expression was calculated from four independent experiments. #*p* < 0.05 with respect to stimulated untreated samples (0 μM)

In both populations, increasing doses of the inhibitors induced a clear trend toward a decrease in the percentage of IFN-γ^+^ and granzyme B^+^ cells (Fig. 4a, b). However, only the diminution of IFN-γ^+^ cells in BEZ235 (5–10 μM) treated samples, both among CD4^+^ and CD8^+^ populations, was significant. Similar results were observed regarding the percentage of CD25^+^ cells among CD8^+^ population, being significantly reduced only in the case of BEZ235 10 μM. On the contrary, the percentage of CD25^+^ cells among CD4^+^ population remained elevated (Fig. 4c), although median fluorescence intensity (MFI) of CD25 was reduced with both inhibitors (Fig. 4d).

In addition, T cells were classified into different maturation subsets based on the expression of CD27 and CD45RA [17]: naïve (CD45RA^+^CD27^+^), early effector (T_EE_; CD45RA^+^CD27^high^), central memory (T_CM_; CD45RA^-^CD27^+^), effector memory (T_EM_; CD45RA^-^CD27^−^), and effector/TEMRA (effector/terminally differentiated effector memory CD45RA^+^ cells; T_E/T_; CD45RA^+^CD27^−^) T cells. The effect of both inhibitors on the different T cell subsets was analyzed.

**Fig. 5:**
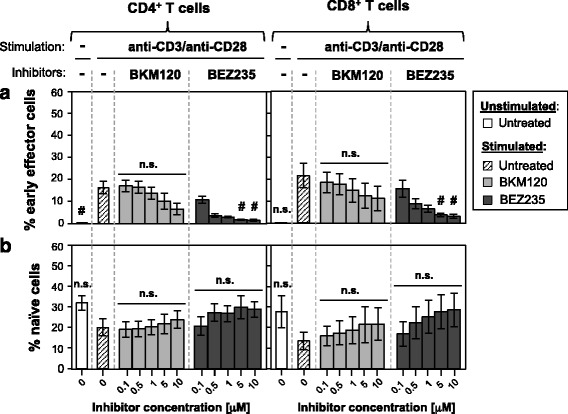
Effect of BKM120 and BEZ235 on the percentage of T cell maturation subsets. Percentage of **a** early effector and **b** naïve cells among CD4^+^ and CD8^+^ cells unstimulated or stimulated in the presence of different concentrations of BKM120 or BEZ235. Mean ± SEM of five different experiments. #*p* < 0.05 with respect to stimulated untreated samples (0 μM); *n.s.* non-significant differences with respect to stimulated untreated samples (0 μM)

Stimulation in the absence of treatment gave rise, among both CD4^+^ and CD8^+^ T cells, to a population of T_EE_ cells, which was significantly reduced in the presence of BEZ235 (Fig. 5a). By contrast, stimulation induced a non-significant decrease in the percentage of naïve cells, which was reversed by the addition of the inhibitors (Fig. 5b).

The percentage of the other subpopulations hardly changed in the presence of the drugs, except the percentage of T_CM_ cells among CD4^+^ population, which showed a trend to decrease with stimulation, and to recover the value of unstimulated control when the drugs were added (Additional file 1: Figure S1).

Regarding the percentage of CD25, IFN-γ and granzyme B-positive cells in the different CD4^+^ and CD8^+^ T cell maturation subsets, the drugs exerted, in general, a similar effect to that observed in CD4^+^ and CD8^+^ whole populations (Additional file 1: Figure S2 and S3). As an exception, the percentage of granzyme B^+^ cells among T_E/T_ cells remained high in the presence of the inhibitors, although the treatment induced a trend to reduce the intensity of expression of this molecule. Moreover, BEZ235 10 μM reduced it significantly (Additional file 1: Figure S4).

### Effect of BKM120 and BEZ235 on T cell tolerization

Next, we assessed whether the drugs were able to induce anergy on alloreactive T cells without hampering the immune response against pathogens. To address this question, PBMCs were stimulated with allogeneic PBMCs in the presence of BKM120 or BEZ235, and, subsequently, with these allogeneic cells or with CMV-pp65 protein in the absence of drugs.

**Fig. 6:**
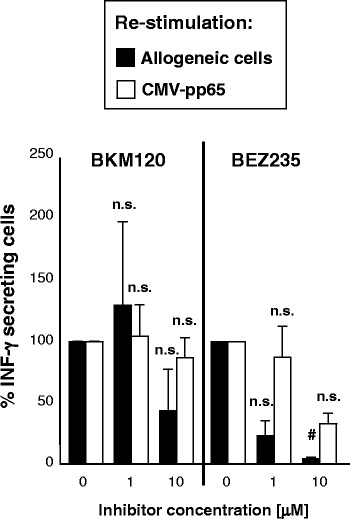
Effect of BKM120 and BEZ235 on T cell tolerization. Percentage of IFN-γ secreting cells among lymphocytes pre-stimulated with allogeneic cells in the presence of different doses of BKM120 or BEZ235 and re-stimulated, in the absence of drugs, with the same allogeneic cells or with CMV-pp65. Every value was normalized to the number of IFN-γ secreting cells that had been pre-stimulated in the absence of drugs (0 μM) and subjected to the corresponding kind of re-stimulation. Results are means + SEM of three independent experiments. #*p* < 0.05 with respect to stimulated untreated samples (0 μM); *n.s.* non-significant differences with respect to stimulated untreated samples (0 μM)

As shown in Fig. 6, BKM120 (10 μM) and BEZ235 (1 μM) induced a non-significant decrease in IFN-γ response to re-stimulation with allogeneic cells, while maintaining a high percentage of IFN-γ secreting cells in response to re-stimulation with CMV-pp65 protein. However, only BEZ235 10 μM induced a significant decrease in IFN-γ secreting cells in response to allogeneic cells.

### Effect of BEZ235 in a murine model of GvHD

Based on the results obtained in vitro, BEZ235 was selected to evaluate its potential utility in GvHD prophylaxis in a murine model.

**Fig. 7:**
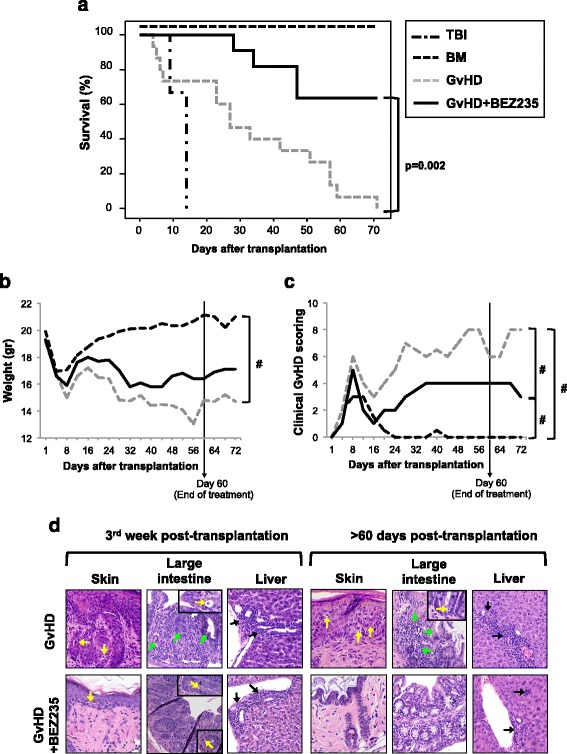
Effect of BEZ235 in a murine model of GvHD. **a** Kaplan–Meier curve representing overall survival of the different experimental groups: TBI (*n* = 4), BM (*n* = 8), GvHD (*n* = 15), and GvHD + BEZ235 (*n* = 11). **b** Evolution of weight loss of transplanted mice (median weight in grams); #*p* < 0.05. **c** GvHD score of transplanted mice (median); #*p* < 0.05. **d** Histopathological analysis of skin, large bowel, and liver samples from the different experimental groups were obtained in the third week after transplantation and once treatment was completed (beyond 60 days after transplantation). Apoptotic bodies (*yellow arrows*), loss of crypts and caliciform cells (*green arrows*) in large bowel, and lymphocytic infiltration in periportal areas (*black arrows*) in the liver are indicated. Original magnification: ×200; insets: ×400

The administration of BEZ235 significantly increased survival (*p* = 0.002) with respect to GvHD untreated mice (Fig. 7a). BEZ235 did not significantly ameliorate the weight loss suffered as a consequence of transplantation (Fig. 7b) but reduced the severity of the other GvHD clinical signs evaluated (Fig. 7c). Histopathological analysis of GvHD target organs was performed at the third week post-transplantation and once treatment was completed (>60 days). Damages in the skin, large intestine, and liver were observed in untreated mice at the third week, and the only mouse that survived beyond day 60 also showed evident GvHD signs in these organs. BEZ235 treatment modestly reduced tissue damage by week 3; however, only mild portal lymphoid infiltrate was observed in BEZ235-treated mice that survived beyond day 60 post-transplantation. The score of GvHD-associated tissue damage in the different groups is summarized in Table 2.

**Table 2:** Score of tissue damage in GvHD target organs in the different treatment groups

		Skin	Large intestine	Liver
Third week post-transplantation	GvHD	2	3	1
	GvHD + BEZ235	2	1	1
>60-day post-transplantation	GvHD	2	2	1
	GvHD + BEZ235	0	0	0,5

## Discussion

In the last decade, numerous class I PI3K inhibitors with various profiles, such as pan-Class I PI3K, isoform-specific PI3K or dual PI3K/mTOR inhibitors, have been developed for clinical applications, especially in the field of oncology. In the current study, we have evaluated the ability of the pan-Class I PI3K inhibitor BKM120 and the dual PI3K/mTOR inhibitor BEZ235 to block T cell activation and shown for the first time the potential utility of BEZ235 in the context of allo-HSCT.

Although largely demonstrated on tumor cells [18, 19], the antiproliferative effect of these drugs on T cells had been scantily evaluated [11, 20]. We have confirmed the ability of BKM120 and BEZ235 to inhibit activated T cell proliferation and, in the case of BEZ235, we have shown for the first time the induction of cell cycle arrest in G0/G1 phase.

Cell proliferation is strongly associated with PI3K/AKT/mTOR pathway, since components such as AKT, RPS6 and 4E-BP1 drive the synthesis and activity of cell cycle-related proteins [21–26]. In this regard, we have shown that the degree of T cell proliferation correlated to phosphorylation levels of AKT, 4E-BP1 and RPS6, and that simultaneous inhibition of PI3K and mTOR was effective at lower concentrations than PI3K inhibition alone.

On the other hand, the inhibitors reduced not only RPS6 and 4E-BP1 phosphorylation, but also their expression. It has been reported that the expression of ribosomal protein genes, such as RPS6, is cell cycle-dependent and, therefore, levels of RPS6 remain low during the G0 phase [27], as we observed in resting T cells. Thus, the decrease of RPS6 expression could be linked to the antiproliferative effect of the inhibitors. Regarding 4E-BP1, it has been shown that its expression is downregulated by the activity of MAPKs ERK and p38 [16]. Interestingly, both drugs induced an increase in p38 phosphorylation, that could contribute to the 4E-BP1 expression decrease. However, as we had previously shown for BKM120, neither of the drugs enhanced ERK phosphorylation on stimulated T cells [11].

On the other hand, BKM120 and BEZ235 induced a dose-dependent decrease in Th1/Th2 cytokine secretion, according to studies performed with PI3K [10, 12] and mTOR inhibitors [28, 29]. Once more, BEZ235 was more potent than BKM120 at intermediate doses. This could be due to the direct effect of BEZ235 on mTOR kinase, which regulates the activity of T-bet and GATA-3, key transcription factors in Th1/Th2 cytokine production [5, 6, 30]. Nevertheless, despite an IL-2 decrease at low doses of BEZ235, concentrations ≥1 μM led to a tendency to recover IL-2 secretion. Probably, the potent inhibition of PI3K/mTOR signaling by BEZ235 leads to upregulation of other pathways that drive IL-2 synthesis, such as Ca^2+^/calcineurin/NFAT, NF-κB or RAS pathways. In this sense, it is known that after T cell stimulation, initial high IL-2 production is followed by its decline, due to the repression of NF-κB and NFAT by T-bet activity [31, 32]. Therefore, mTORC1 inhibition exerted by BEZ235 would decrease mTORC1-dependent expression of T-bet [5], leading to IL-2 secretion maintenance. In addition, inhibition of mTOR and PI3K can increase RAS signaling as a compensatory mechanism, leading to an increase in ERK phosphorylation [33, 34]. However, we have not observed a higher ERK phosphorylation in treated T cells. In any case, the increase in IL-2 levels does not induce an increase in T cell proliferation, probably due to the downregulation of IL-2 receptor alpha chain (CD25) observed in the presence of the drug.

Regarding IFN-γ and granzyme B, their expression is dependent on T-bet and EOMES [35, 36], which are regulated by mTORC1 and mTORC2 activity [37]. Accordingly, intracellular expression of these molecules decreased in the presence of BKM120 and, specially, of BEZ235.

With respect to apoptosis, we have observed that BEZ235 does not induce significant apoptosis, neither among resting nor among stimulated T lymphocytes, as we had previously shown for BKM120 [11, 12]. Moreover, caspase 3 activation, generally considered to be an apoptosis indicator, decreased with the addition of BKM120 and BEZ235. This could indicate that the drugs reduce apoptosis in activated lymphocytes. However, it should be noted that the inhibitors prevent activation and, therefore, activation-induced cell death [38]. In addition, it has been reported that T cell activation induces cleavage of caspase 3 in the absence of apoptosis [39, 40], what could be reversed by activation inhibition achieved by the drugs. In any case, neither BKM120 nor BEZ235 seems to induce apoptosis in stimulated T cells. Other authors have also shown limited toxicity of BKM120 and BEZ235 toward normal PBMCs [41–43].

On the other hand, standard GvHD prophylaxis is associated with high risk of life-threatening opportunistic infections [44]. Thus, GvHD prophylactic strategies should induce tolerance of alloreactive T cells but maintain an adequate immune response against pathogens, such as cytomegalovirus (CMV). Previous studies have shown that mTORC1 inhibition with rapamycin can induce T cell anergy in vitro [45, 46] and reduce GvHD incidence [8] as well as CMV reactivation [47] after allo-HSCT. In addition, we had proved that mTOR inhibition during allogeneic PBMC stimulation induced tolerance of alloreactive T cells while preserving immune response against CMV [48]. In the present study, we have shown that not only mTOR inhibition but also PI3K inhibition provides these results.

In general, in vitro studies have shown that BEZ235 exerts a strong inhibitory effect at lower concentrations than BKM120, what is logical since BEZ235 inhibits more potently the different PI3K isoforms and simultaneously targets mTOR. However, it is worthy to note that the concentrations above which BKM120 achieves similar inhibition to BEZ235 (≥5 μM) are precisely those in which it drastically decreases phosphorylation of mTOR targets (4EBP1, RPS6, AKT S473), that is, between 1 and 10 μM, as shown by Western blot. Moreover, BKM120 non-specific activity against mTOR has been described at concentrations greater than 2 μM [49]. Thus, it is possible that the potent effect observed at high concentrations of BKM120 is due to a direct effect on mTOR as well. This would indicate that simultaneous targeting of PI3K and mTOR achieve a better inhibitory capacity than PI3K inhibition alone. On the other hand, we had previously observed that dual mTORC1/mTORC2 targeting exerts a more potent T cell inhibition than mTORC1 blockade [48], and similar to PI3K/mTOR targeting. Thus, we could conclude that to effectively block PI3K/AKT/mTOR pathway in T cells, we should block, at least, both mTORC1 and mTORC2 complexes, accompanied or not by PI3K inhibition.

Importantly, BEZ235 reduced more effectively than BKM120 the percentage of T_EE_ cells, which arise from naïve T cells after stimulation [50]. This ability to prevent naïve T cell activation is important, given the key role played by this T cell population in GvHD induction [51, 52].

Finally, we selected BEZ235 to evaluate its utility on GvHD prophylaxis. BEZ235 significantly improved mice survival and ameliorated GvHD-associated signs. On the other hand, it is probable that the damage observed in BEZ235-treated animals is not entirely caused by GvHD, but possibly to drug-induced toxicity as well. In this sense, it has been reported that the use of PI3K/AKT/mTOR pathway inhibitors is associated with metabolic disorders and damages in skin, liver or gastrointestinal mucosa [53]. Thus, the tissue damage observed at third week post-transplantation could be due, at least in part, to a toxic effect of the drug difficult to discern from GVHD-induced damage in early post-transplant phases [54, 55]. In addition, BEZ235 administration in murine tumor models induces a weight loss associated with a lower food intake [56]. Thus, further studies are warranted to optimize BEZ235 dosing in order to reduce toxicity.

Our results show the ability of novel PI3K inhibitors to control T cell activation and confirm their potential utility as a therapeutic alternative in GvHD management. Nevertheless, two questions must be addressed. On the one hand, the concern arises about their potential negative effect on anti-leukemia T cells, which would have a negative impact on relapse incidence. Previous [42, 43, 57–59] and future studies analyzing their antitumor activity on hematologic malignancies will help to elucidate if their administration could counterbalance this negative effect and reduce the incidence of relapse. On the other hand, it should be noted that the overall in vivo impact of PI3K inhibitors on T cells will also depend on their effect on other immune cells that modulate or contribute to T cell activation, such as antigen-presenting cells. In this sense, both pro- and anti-inflammatory effects of PI3K inhibitors on monocytes and DCs have been described [60–62]. In our hands, the use of PI3K inhibitors in total PBMC cultures induced an immunosuppressive effect on T lymphocytes similar to that observed in isolated T cell cultures (present work and data not shown).

## Conclusions

PI3K inhibitors hold promise for the treatment of T cell-mediated diseases, in general, and in particular of GVHD. Their ability to hamper T cell function together with their potential anti-leukemia effect turn the use of drugs that target PI3K/AKT/mTOR pathway into a promising approach in the context of allo-HSCT.

## Additional file

Additional file 1: Figures S1-S4.
**Figure S1.** Effect of BKM120 and BEZ235 on the percentage of central memory, effector memory, and effector/TEMRA T cells. Percentage of central memory, effector memory and effector/TEMRA cells among CD4^+^ and CD8^+^ cells unstimulated or stimulated in the presence of different concentrations of BKM120 or BEZ235. Mean ± SEM of five different experiments. # *p* < 0.05 with respect to stimulated untreated samples (0 μM). **Figure S2.** Effect of BKM120 and BEZ235 on the phenotype of different CD4^+^ T cell maturation subsets. Percentage of cells expressing CD25, IFN-γ, and granzyme B among different CD4^+^ T cell maturation subsets, in samples unstimulated or stimulated in the presence of different concentrations of BKM120 or BEZ235. Mean + SD of five different experiments. # *p* < 0.05 with respect to stimulated untreated samples (0 μM). **Figure S3.** Effect of BKM120 and BEZ235 on the phenotype of different CD8^+^ T cell maturation subsets. Percentage of cells expressing CD25, IFN-γ, and granzyme B among different CD8^+^ T cell maturation subsets, in samples unstimulated or stimulated in the presence of different concentrations of BKM120 or BEZ235. Mean + SD of five different experiments. # *p* < 0.05 with respect to stimulated untreated samples (0 μM). **Figure S4.** Effect of BKM120 and BEZ235 on granzyme B expression by effector/TEMRA T cells. Median fluorescence intensity (MFI) of granzyme B among effector/TEMRA CD4^+^ and CD8^+^ T cells unstimulated or stimulated in the presence of different concentrations of BKM120 or BEZ235. Mean + SD of four different experiments. # *p* < 0.05 with respect to stimulated untreated samples (0 μM). (PDF 334 kb)

## Abbreviations

7AAD: 7-amino-actinomycin D
allo-HSCT: Allogeneic hematopoietic stem cell transplantation
CBA: Cytokine Cytometric Bead Array
CMV: Cytomegalovirus
GvHD: Graft-versus-host disease
MAPKs: Mitogen-activated protein kinases
PBMCs: Peripheral blood mononuclear cells
PI3K/AKT/mTOR: Phosphatidylinositol 3-kinase/AKT/mammalian target of rapamycin
TBI: Total body irradiation
T_CM_: Central memory T cells
T_E/T_: Effector/TEMRA (effector/terminally differentiated effector memory CD45RA^+^ cells) T cells
T_EE_: Early effector T cells
T_EM_: Effector memory T cells
