# Supplementary material for: Targeting of PI3K/AKT/mTOR pathway to inhibit T cell activation and prevent graft-versus-host disease development
Source: J Hematol Oncol. 2016 Oct 20;9:113. doi: 10.1186/s13045-016-0343-5 (PMC5072323; doi:10.1186/s13045-016-0343-5)
Supplement: Additional file 1: Figures S1-S4. — Figure S1. Effect of BKM120 and BEZ235 on the percentage of central memory, effector memory, and effector/TEMRA T cells. Percentage of central memory, effector memory and effector/TEMRA cells among CD4+ and CD8+ cells unstimulated or stimulated in the presence of different concentrations of BKM120 or BEZ235. Mean ± SEM of five different experiments. # p < 0.05 with respect to stimulated untreated samples (0 μM). Figure S2. Effect of BKM120 and BEZ235 on the phenotype of different CD4+ T cell maturation subsets. Percentage of cells expressing CD25, IFN-γ, and granzyme B among different CD4+ T cell maturation subsets, in samples unstimulated or stimulated in the presence of different concentrations of BKM120 or BEZ235. Mean + SD of five different experiments. # p < 0.05 with respect to stimulated untreated samples (0 μM). Figure S3. Effect of BKM120 and BEZ235 on the phenotype of different CD8+ T cell maturation subsets. Percentage of cells expressing CD25, IFN-γ, and granzyme B among different CD8+ T cell maturation subsets, in samples unstimulated or stimulated in the presence of different concentrations of BKM120 or BEZ235. Mean + SD of five different experiments. # p < 0.05 with respect to stimulated untreated samples (0 μM). Figure S4. Effect of BKM120 and BEZ235 on granzyme B expression by effector/TEMRA T cells. Median fluorescence intensity (MFI) of granzyme B among effector/TEMRA CD4+ and CD8+ T cells unstimulated or stimulated in the presence of different concentrations of BKM120 or BEZ235. Mean + SD of four different experiments. # p < 0.05 with respect to stimulated untreated samples (0 μM). (PDF 334 kb) [file 13045_2016_343_MOESM1_ESM.pdf]

Supplementary Information

Figure S1. Effect of BKM120 and BEZ235 on the percentage of central memory, effector memory and effector/TEMRA T cells. Percentage of central memory, effector memory and effector/TEMRA cells among $CD4^+$ and $CD8^+$ cells unstimulated or stimulated in the presence of different concentrations of BKM120 or BEZ235. Mean ± SEM of five different experiments. **#** $p<0.05$ with respect to stimulated untreated samples (0 μM).

Figure S2. Effect of BKM120 and BEZ235 on the phenotype of different $CD4^+$ T cell maturation subsets. Percentage of cells expressing CD25, IFN-γ and granzyme B among different $CD4^+$ T cell maturation subsets, in samples unstimulated or stimulated in the presence of different concentrations of BKM120 or BEZ235. Mean +SD of five different experiments. **#** $p<0.05$ with respect to stimulated untreated samples (0 μM).

Figure S3. Effect of BKM120 and BEZ235 on the phenotype of different $CD8^+$ T cell maturation subsets. Percentage of cells expressing CD25, IFN-γ and granzyme B among different $CD8^+$ T cell maturation subsets, in samples unstimulated or stimulated in the presence of different concentrations of BKM120 or BEZ235. Mean +SD of five different experiments. **#** $p<0.05$ with respect to stimulated untreated samples (0 μM).

Figure S4. Effect of BKM120 and BEZ235 on granzyme B expression by effector/TEMRA T cells. Median Fluorescence Intensity (MFI) of granzyme B among effector/TEMRA $CD4^+$ and $CD8^+$ T cells unstimulated or stimulated in the presence of different concentrations of BKM120 or BEZ235. Mean +SD of four different experiments. **#** $p<0.05$ with respect to stimulated untreated samples (0 μM).

Figure S1.

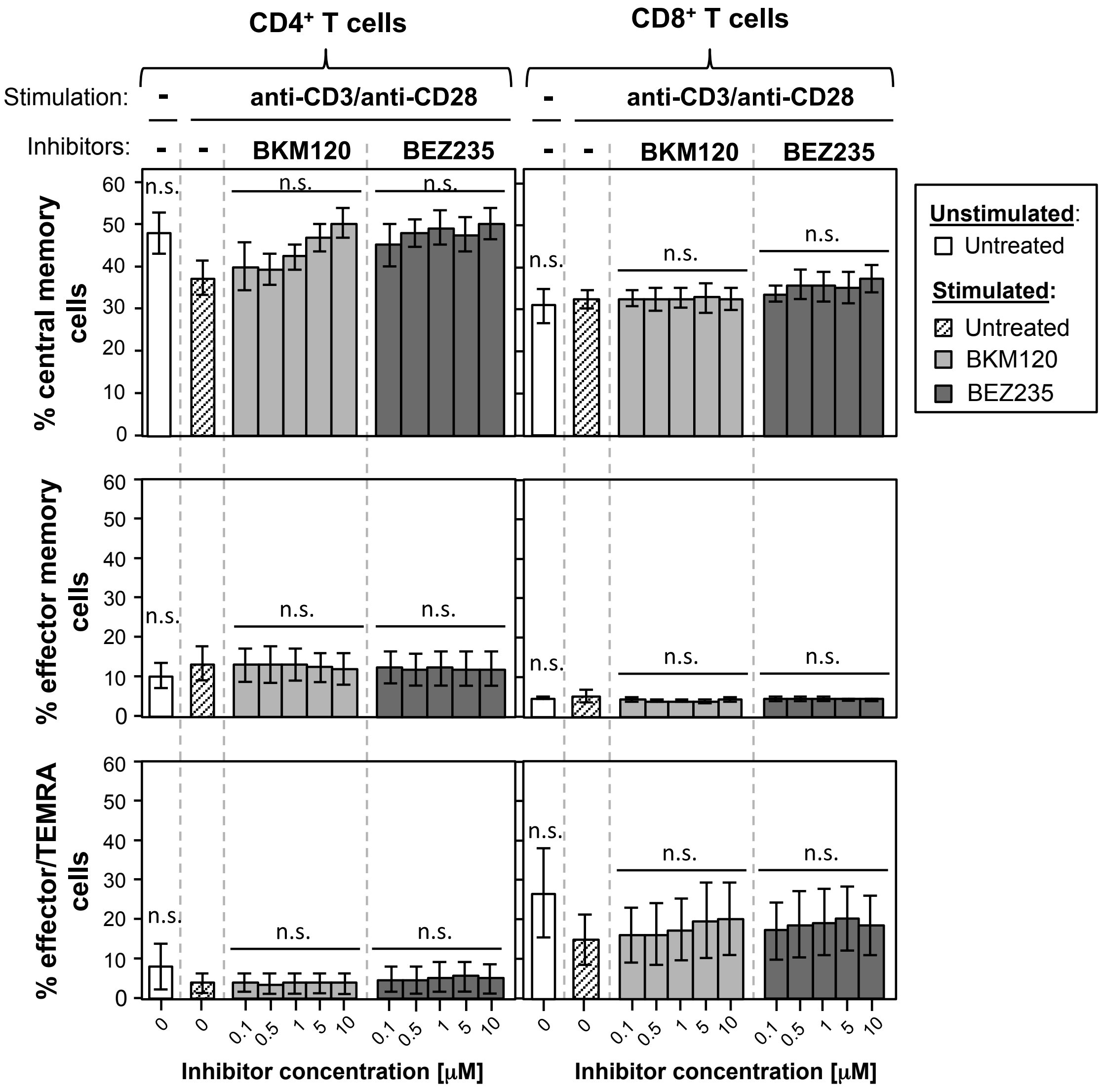
CD4⁺ T cells
CD8⁺ T cells
Stimulation:
-
anti-CD3/anti-CD28
Inhibitors:
BKM120
BEZ235
% central memory cells
% effector memory cells
% effector/TEMRA cells
n.s.
Unstimulated:
Untreated
Stimulated:
Untreated
BKM120
BEZ235
Inhibitor concentration [μM]

Figure S2.

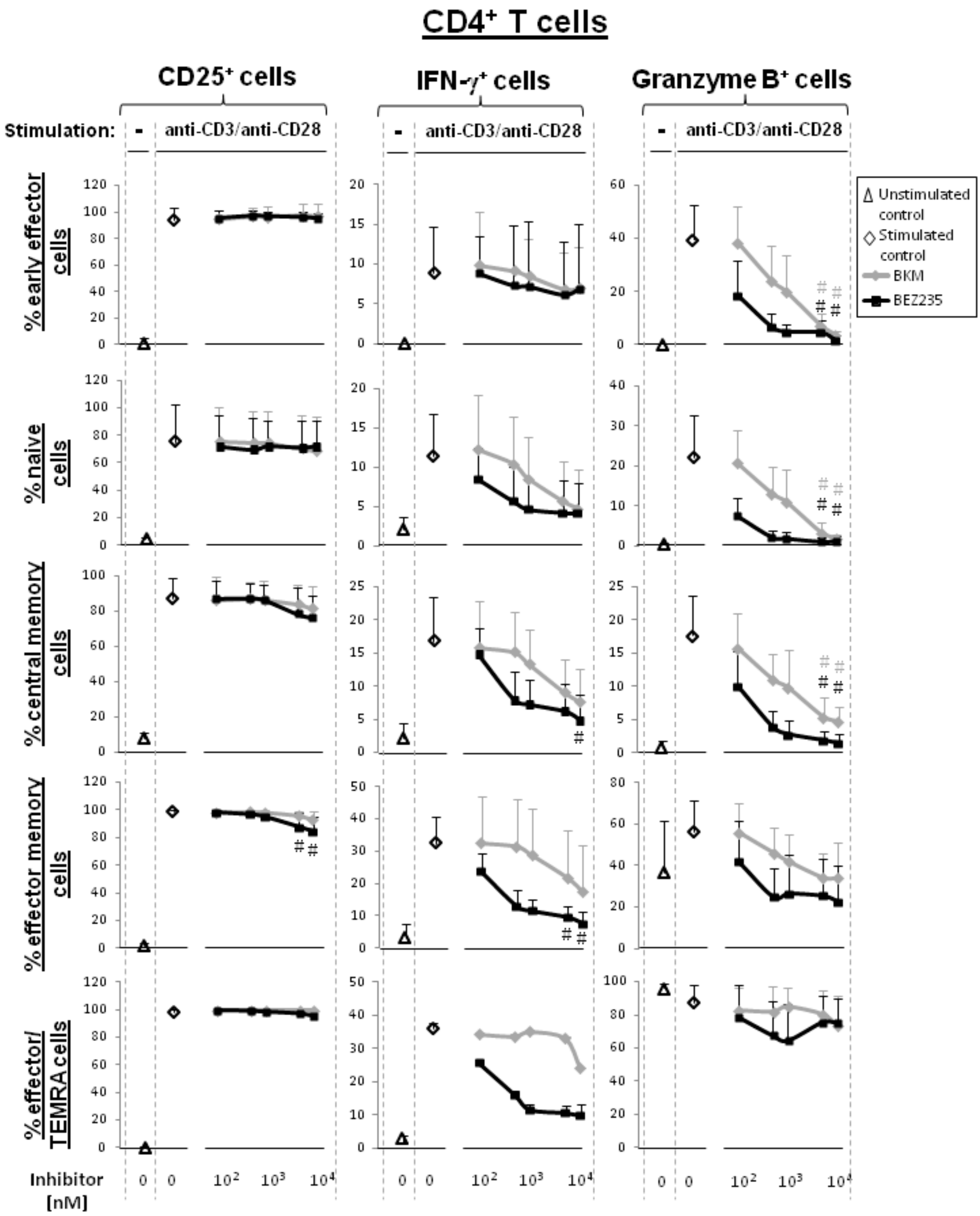
CD4+ T cells
CD25+ cells
IFN-γ+ cells
Granzyme B+ cells
Stimulation:
-
anti-CD3/anti-CD28
% early effector cells
% naive cells
% central memory cells
% effector memory cells
% effector/ TEMRA cells
Unstimulated control
Stimulated control
BKM
BEZ235
Inhibitor [nM]
0
0
10^2
10^3
10^4
#

Figure S3.

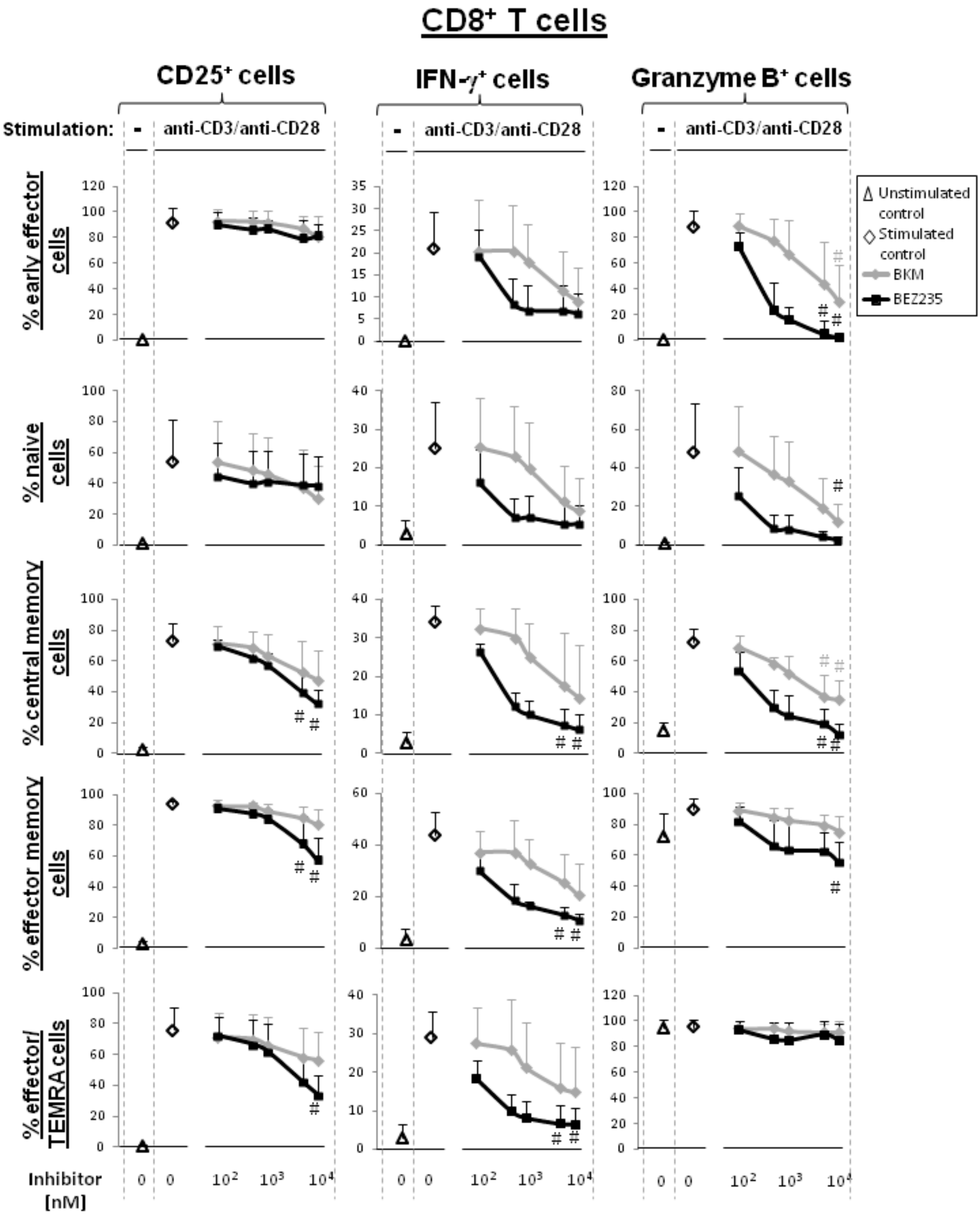
CD8⁺ T cells
CD25⁺ cells
IFN-γ⁺ cells
Granzyme B⁺ cells
Stimulation:
-
anti-CD3/anti-CD28
% early effector cells
% naive cells
% central memory cells
% effector memory cells
% effector/ TEMRA cells
Unstimulated control
Stimulated control
BKM
BEZ235
Inhibitor [nM]
0
10²
10³
10⁴

FIGURE S4.

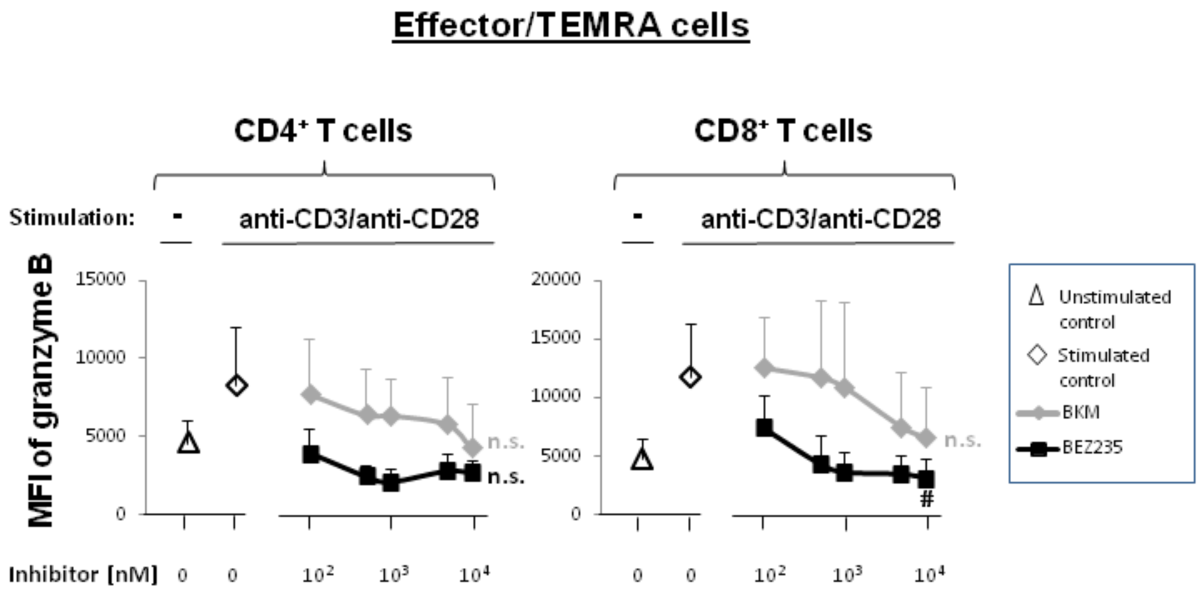

Effector/TEMRA cells
CD4+ T cells
CD8+ T cells
Stimulation:
-
anti-CD3/anti-CD28
MFI of granzyme B
15000
10000
5000
0
20000
n.s.
#
Inhibitor [nM]
0
10²
10³
10⁴
Unstimulated control
Stimulated control
BKM
BEZ235
